# CD38 in the pathobiology of cutaneous T-cell lymphoma and the potential for combination therapeutic intervention

**DOI:** 10.1038/s41375-025-02551-4

**Published:** 2025-03-08

**Authors:** Colleen Isabelle, Amy Boles, Kathleen McConnell, Robyn Keller, Rachel Burzinski, Zachary Hutchins, Giulia Calabretto, Lara Cheslow, Jonathan Xu, Nitin Chakravarti, Pierluigi Porcu, Neda Nikbakht, Anjali Mishra

**Affiliations:** 1 Department of Medical Oncology, Sidney Kimmel Cancer Center, Thomas Jefferson University, Philadelphia, PA USA; 2 Department of Neuroscience, Thomas Jefferson University, Philadelphia, PA USA; 3 Department of Dermatology and Cutaneous Biology, Sidney Kimmel Cancer Center, Thomas Jefferson University, Philadelphia, PA USA

**Keywords:** T-cell lymphoma, Targeted therapies

## Abstract

Cutaneous T-Cell Lymphoma (CTCL) is a non-Hodgkin’s lymphoma involving malignant skin-homing T-cells, characterized by variable severity and limited treatment options. Our study shows that patient samples and derived cell lines express CD38 on CTCL cells, and αCD38 antibodies effectively target CD38 in a mouse model. In vivo αCD38 antibody treatment led to the loss of CD38 expression in residual tumor cells, highlighting the need for innovative strategies to improve CTCL outcomes despite the CD38 loss in residual tumor cells. To investigate the role of CD38 in CTCL pathology, we used CRISPR-Cas9 to create CD38-deficient (CD38^KO^) CTCL cells. These CD38^KO^ cells showed higher expression of oncogenes *B-catenin*, *TCF7*, and *BCL6*, along with reduced migration. Elevated NAD+ levels in CD38^KO^ cells increased cellular respiration after CD38 inhibition in CD38^WT^ cells. In vivo, CD38^KO^ cell transplants led to more aggressive tumors, likely due to elevated β-catenin, Bcl6, and Tcf-1 signaling. Prior research in multiple myeloma showed αCD38 antibody efficacy relies on CD38 expression. We discovered that panobinostat, a histone deacetylase inhibitor, increased surface CD38 expression in CTCL cells dose-dependently. Combining panobinostat with αCD38 antibody in a CTCL mouse model significantly improved survival compared to the antibody alone, underscoring CD38’s therapeutic potential in CTCL.

**Figure:**
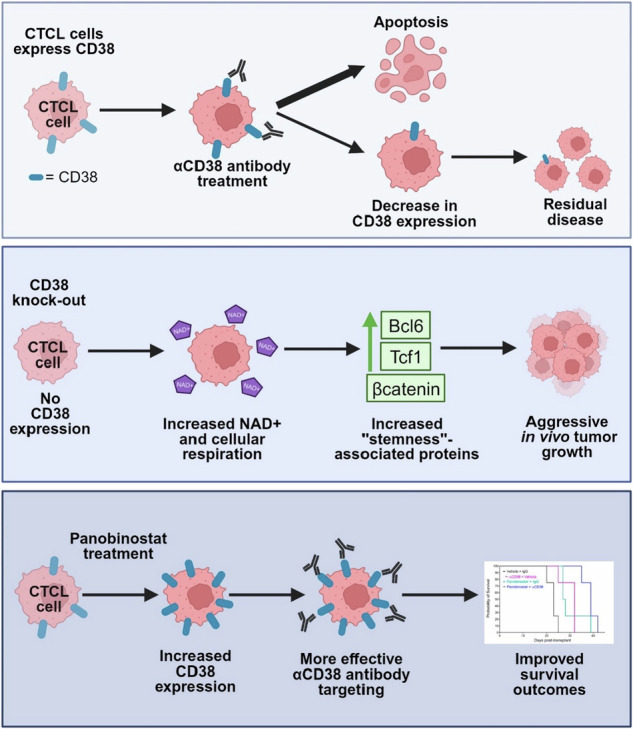
CD38 is expressed in CTCL cells and can be targeted with αCD38 antibody. αCD38 antibody treatment leads to a significant reduction in CTCL cells, while residual cells lose CD38 expression. Knocking out CD38 from CTCL cells leads to increases in intracellular NAD+ and increased cellular respiration. Additionally, CD38^KO^ cells have increased protein levels of β-catenin, Tcf1 (encoded by *TCF7*), and Bcl6. CD38^KO^ CTCL cells grow more aggressively in vivo than CD38^WT^ CTCL cells. Treating CTCL cells with panobinostat increases CD38 expression. A dual combination treatment of panobinostat and αCD38 antibody in a mouse model of CTCL improved survival outcomes compared to αCD38 antibody treatment alone. (Figure made with Biorender.com).

CD38 is expressed in CTCL cells and can be targeted with αCD38 antibody. αCD38 antibody treatment leads to a significant reduction in CTCL cells, while residual cells lose CD38 expression. Knocking out CD38 from CTCL cells leads to increases in intracellular NAD+ and increased cellular respiration. Additionally, CD38^KO^ cells have increased protein levels of β-catenin, Tcf1 (encoded by *TCF7*), and Bcl6. CD38^KO^ CTCL cells grow more aggressively in vivo than CD38^WT^ CTCL cells. Treating CTCL cells with panobinostat increases CD38 expression. A dual combination treatment of panobinostat and αCD38 antibody in a mouse model of CTCL improved survival outcomes compared to αCD38 antibody treatment alone. (Figure made with Biorender.com).

## Introduction

Cutaneous T-cell Lymphoma (CTCL) is a group of rare mature T-cell neoplasms with limited treatment options and a broad spectrum of prognoses depending on disease severity [1]. Treatment relapse and resistance are a common problem. The only potential curative therapy option is allogeneic stem cell transplant, which confers significant risks and still has a limited median survival of only 9 months [2]. While early forms of CTCL are often indolent, approximately 30% of patients will experience disease progression, which occurs when tumor cells are found in the blood and lymph nodes [1]. Additionally, some patients will initially present with advanced stage disease. Survival rates for severe forms of CTCL are grim, with most patients only surviving 2–5 years post diagnosis [1]. Additional research is needed into novel targets for therapeutic intervention to improve these outcomes.

CD38 is a cell surface marker of interest we have previously shown to be upregulated in mature T-cell neoplasms [3]. CD38 is a multifunctional molecule that can act as both a hydrolyzing enzyme of NAD+ as well as a signaling receptor and is dysregulated in many hematological malignancies [4, 5]. The role of CD38 in CTCL pathology is still unknown. While two CD38-specific antibodies are approved for clinical use in relapsed/refractory multiple myeloma, it’s unclear if this approach benefits CTCL patients. Our previous study demonstrated the preclinical efficacy of αCD38 antibody treatment in vivo using two mouse models of mature T-cell neoplasms, including one with the Hut-78 cell line from a severe CTCL patient. αCD38 antibody treatment significantly reduced tumor cell burden and improved survival outcomes in CTCL mice compared to a vehicle control [3]. However, the effect of the αCD38 antibody was not curative and residual disease remained. Previous evidence from MM has shown decreases in CD38 expression on MM cells post αCD38 antibody exposure and combination drug treatment methods have been employed to help combat this method of tumor cell escape [6, 7]. Some of the pharmaceutical agents that have shown efficacy in upregulating CD38 expression in MM are all-trans retinoic acid, azacytidine, and histone deacetylase inhibitors (HDACi) [8–10]. The effects of αCD38 antibody on CD38 expression in CTCL and the possibility of pharmacologically increasing CD38 expression in these cells are unexplored. Furthermore, the synergy between a CD38-upregulating drug and αCD38 antibody treatment in CTCL has not been investigated.

In our study, we identified a subset of CTCL cells resistant to CD38 antibody treatment in mice, investigating its impact on post-treatment tumor growth. We revealed the unique characteristics of CD38-negative CTCL cells, shedding light on how this phenomenon affected tumor progression after treatment. Our findings reveal elevated CD38 expression in CTCL, making it a viable target for αCD38 antibody therapy to reduce tumor burden. Despite αCD38 resistance reported in MM due to decreased CD38 expression, our exploration of reduced CD38 expression in CTCL shows it promotes aggressive in vivo cell proliferation. Notably, a combination therapy with HDACi panobinostat and αCD38 antibody effectively reduces tumor mass and enhances survival rates in a CTCL mouse model. These results emphasize the need for further exploration into CD38’s role in CTCL pathology and suggest αCD38 antibody as a potential therapeutic option for CD38-positive CTCL patients.

## Methods

### Cell culture and flow cytometry

We obtained CTCL cell lines (HH, H9, Hut78, and Hut102) from the American Type Culture Collection (ATCC; MD, USA) and followed their recommended growth conditions. Regular authenticity verification and mycoplasma contamination testing were performed. Flow cytometry antibodies (CD4 APC-Cy7, CD38 PE-Cy7, CD45 BV421, CXCR4 BV421 and isotype control PE) were purchased from BD Biosciences. See supplementary material for additional methods.

### Engineered cell lines

CD38 knockouts were generated in CTCL cell lines (HH, Hut78, and H9) using CRISPR-Cas9 with multi-guide sgRNA Synthego. Genetic modification was done with specific primers (Synthego kit; primers: U*G*C*UCGCGGUGGUCGUCCCG + Synthego modified EZ scaffold and G*A*C*GGUCUCGGGAAAGCGCU + Synthego modified EZ scaffold), using the Amaxa Nucleofector I kit. Firefly luciferase-GFP lentivirus was introduced, followed by cell sorting to obtain highly pure CD38^WT^ and CD38^KO^ cell populations (purity >99%) using BD FACSMelody and FACSDiva software. Sorted cells were cultured and monitored for purity and GFP expression using flow cytometry to ensure the desired phenotypes and consistent GFP expression levels between CD38^WT^ and CD38^KO^ lines.

### Gene expression analysis

We examined CD38 expression in human skin biopsies using data from GEO Dataset Accession Number: GSE143382 [11]. RNA profiling from this dataset was analyzed for *CD38* expression using Prism software. Additionally, Genewiz conducted RNA sequencing to support the gene expression analysis. A single cell RNA-seq gene expression matrix was analyzed, encompassing skin samples from five advanced CTCL patients, two individuals with classical MF, and four healthy controls. This data was retrieved from the NCBI Gene Expression Omnibus (GEO) under accession numbers GSE128531 [12], GSM5280111 [13], GSE165623 [14]. See supplementary materials for additional information.

### CTCL xenograft mouse models

All animal experiments were conducted in immunodeficient NOD Rag−/−γc−/− (NRG) mice with Institutional Animal Care and Use Committee approval. Mice received 2 million CTCL cells via either intravenous or subcutaneous engraftment with age (between 6–12 weeks) and sex matched groups (both males and females). Engraftment and tumor progression were monitored using luciferin substrate (150 mg/kg) and In Vivo Imaging System (IVIS; Perkins-Elmer). Tumor burden was quantified via luminescent pixel intensity in Living Image software. Daratumumab (DARZALEX Faspro) and IgG control (Selleckchem) were administered subcutaneously at 100 mg/kg and 0.8 mg/kg respectively. IgG isotype dosage was titrated and showed no significant difference in outcome between 100 mg/kg and 0.8 mg/kg groups (Supplementary Fig. 1). Panobinostat was administered subcutaneously, dissolved in 2% DMSO, 48% PEG300, 2% Tween80 and 48% ddH_2_O. Treatments were administered as described in the timeline. Imaging was performed regularly until study endpoints were reached. See supplementary materials for additional information.

### Metabolism assays

NAD+ and NADH levels were assessed using the NAD + /NADH-Glo Assay kit (Promega, #G9071). Cellular respiration was measured with the XFe24 Seahorse Assay system (Agilent, Santa Clara, CA). The XFe24 cell culture microplates were prepared with Poly-D-Lysine coating, and tumor cells were plated in Seahorse XF RPMI media with specific supplements. The Seahorse XF Mito Stress Test Kit reagents (Oligomycin, FCCP, Rotenone/AA) were added at specified concentrations. After the assay, cells were lysed for protein quantification and normalization using Bio-Rad Protein Assay Dye Reagent Concentrate (BioRad, #5000006). The CD38 inhibitor 78c compound (Selleckchem, #S8960) was used at a concentration of 0.5uM overnight.

### Cell migration assay

H9 CD38^WT^ and CD38^KO^ cells were plated in the upper well of a 8uM transwell migration assay chamber (Costar REF#3422) in complete media. In the lower chamber there was either 100 ng/mL CXCL12/SDF-1a (Thermofisher; Cat#300-28 A) chemoattractant, or 0.1%BSA in PBS control. Plate was incubated at 37 °C and 5% CO_2_ overnight and migrated cells were quantified from the bottom chamber.

### Sample size and statistical methods

See supplementary materials.

## Results

### CD38 is expressed by CTCL tumor cells

**Fig. 1:**
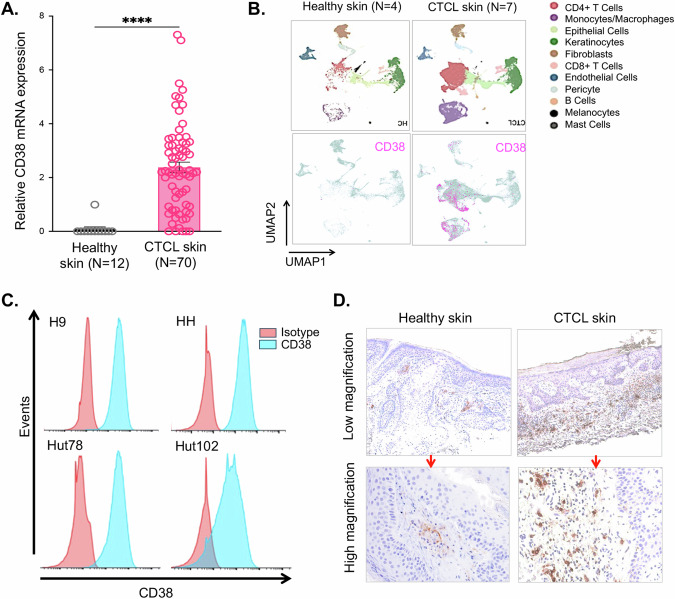
CD38 expression in CTCL cells. **A** *CD38* expression in CTCL was assessed using data from Nielsen et al. 2021 (GSE143382), comparing relative *CD38* expression in skin biopsy samples from CTCL patients (*N* = 70) to healthy donors (*N* = 12) (log fold change 4.8; *p* < 0.0001 by Mann–Whitney test). **B** Single-cell RNA sequencing analysis from previously published datasets (GSE128531, GSM5280111, GSE165623) [12–14] compared *CD38* cell expression in cells from healthy human skin (*N* = 4) to CTCL patient skin (*N* = 7). **C** CD38 expression in CTCL cell lines H9, HH, Hut78, and Hut102 was analyzed by flow cytometry. **D** Formalin-fixed, paraffin-embedded (FFPE) skin biopsies from CTCL patients (*N* = 6) and healthy donor skin (*N* = 1) were stained for CD38 and imaged at original magnification x4 and x20 using a Cytation5 imager and Gen5 software.

While CD38 expression in skin biopsies and blood samples from patients with refractory/relapsed leukemic CTCL has been established previously [15], our study delves into the analysis of existing datasets concerning RNA expression of *CD38* in CTCL skin samples. Additionally, we investigate how *CD38* expression influences the therapeutic effectiveness of CD38 antibody therapy on neoplastic T-cells. Utilizing previously published microarray data (GSE143382; [11]) comparing CTCL and healthy human skin, our analysis reveals a substantial 4.8 log-fold increase in *CD38* expression among CTCL patients in comparison to healthy controls (*p* < 0.0001 by Mann–Whitney test) (Fig. 1A). These findings were subsequently supported by other published single-cell RNA sequencing analyses conducted on skin/tumor samples from healthy individuals (*N* = 4) and CTCL patients (*N* = 7) [12–14]. Uniform Manifold Approximation and Projection (UMAP) analysis of this data set illustrates an elevated expression of *CD38* in neoplastic cell clusters within the CTCL patient skin cohort when compared to the healthy human skin (Fig. 1B). Intriguingly, there was also a noticeable increase in *CD38* expression within another cluster composed of myeloid immune cells. Additionally, we conducted an examination of CD38 protein expression on four human CTCL cell lines: H9, HH, Hut78, and Hut102 (all acquired from ATCC) by flow cytometry. Notably, all four of these CTCL cell lines exhibited high levels of surface CD38 staining (Fig. 1C). To further validate these findings, we conducted immunohistochemical staining for CD38 to confirm protein expression in skin biopsies from CTCL patients (*N* = 6) and healthy skin. Our results clearly indicate that lesional skin biopsies from CTCL patients displayed increased positive staining for CD38 in comparison to the minimal staining observed in healthy human skin (Fig. 1D). Collectively, these results provide robust support for the increased expression of CD38 in CTCL cells.

### The αCD38 therapy led to a substantial clearance of CTCL cells in vivo

**Fig. 2:**
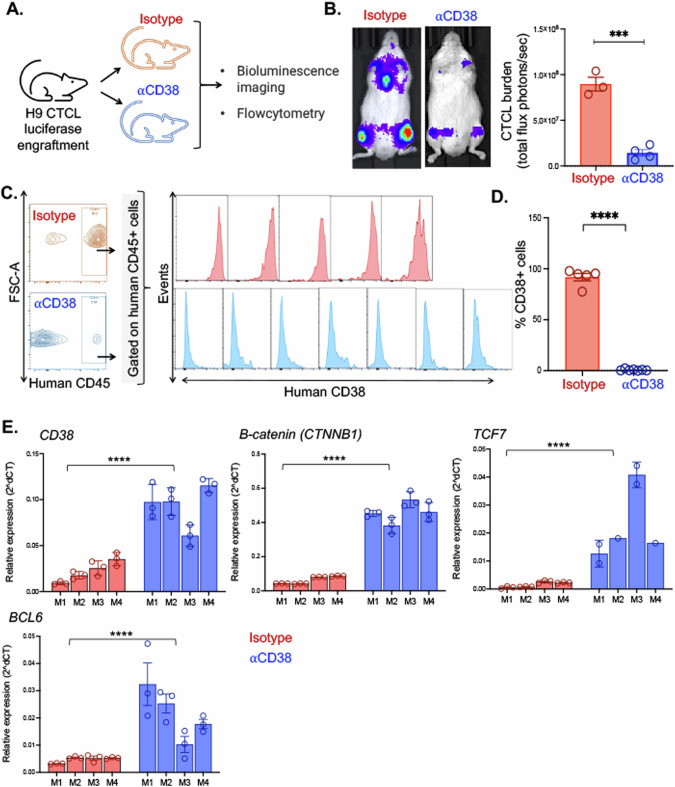
αCD38 antibody treatment reduces CD38 expression and increases stemness genes in CTCL cells. **A** Schematic representation of the experimental design for in vivo testing. CD38+ luciferase expressing CTCL cells (H9 line) were engrafted intravenously in immunodeficient NRG mice which were randomly assigned to treatment conditions with either αCD38 antibody (daratumumab; 100 mg/kg subcutaneously weekly for four weeks) or IgG isotype control (0.8 mg/kg subcutaneously once a week for four weeks). Tumor burden was measured over time using IVIS imaging and quantified using Living Image software. **B** Representative images of mice treated with isotype or αCD38 antibody and quantification of tumor burden total flux (photons/second) at 19 days post-engraftment. αCD38 antibody treatment resulted in an average total flux = 1.4e7 photons/sec (*N* = 4), while IgG average total flux = 9.0e7 photons/sec (*N* = 3); *p* = 0.0002. **C** Flow cytometry analysis of tumor cells isolated from the bone marrow of mice, performed 28 days post-engraftment after three weeks of treatment with either IgG isotype control (*N* = 5) or αCD38 (*N* = 7). Lymphocytes were gated for human CD45+ cells and analyzed for human CD38 signal. **D** Quantification of the percentage of CD45+ tumor cells expressing CD38 based on the data from panel (**C**) (*p* < 0.0001 by unpaired *t* test). **E** qPCR analysis of the relative expression of *CD38*, *B-catenin* (*CTNNB1*), *TCF7*, and *BCL6* genes in CTCL tumor cells isolated from the bone marrow of mice that were treated with either isotype or αCD38 antibody (*p* < 0.0001 in all conditions by 2way ANOVA).

Previously, we demonstrated the preclinical effectiveness of targeting CD38 in mature T-cell neoplasms and we wanted to extend this therapeutic approach to the context of CTCL [3]. To facilitate this investigation, we introduced the firefly-luciferase gene into the H9 CTCL cells, allowing us to monitor these cells using luminescent imaging in living mice. Subsequently, we intravenously transplanted NRG mice with the H9 CTCL luciferase cells and then divided them into two treatment groups: one receiving IgG isotype control and the other receiving αCD38 antibody therapy (Fig. 2A). Over a period of four weeks, we administered treatment to the mice and closely monitored the progression of tumors through IVIS imaging. Notably, the use of daratumumab (αCD38 antibody) had a significant therapeutic impact on CTCL tumors compared to isotype control group, resulting in an impressive 84% reduction in tumor burden (*p* = 0.0002) (Fig. 2B).

### Evidence of the loss of CD38 expression in residual CTCL cells following αCD38 therapy in vivo

The issue of treatment escape and CD38 downregulation following αCD38 antibody treatment has been previously observed in the context of multiple myeloma (MM) when employing this immunotherapeutic targeting strategy [7]. Given the significant effectiveness of αCD38 antibody therapy in reducing tumor burden, albeit without achieving a complete cure, we endeavored to investigate whether residual CTCL tumor cells following treatment displayed diminished CD38 expression in comparison to those that persisted after IgG isotype treatment. To address this question, we collected bone marrow samples from mice with engrafted CTCL tumors that had undergone three weeks of treatment with either αCD38 antibody or IgG isotype control. Subsequently, we stained these cells with human CD45 and human CD38 antibodies for flow cytometry analysis. Lymphocytes were gated on human CD45+ cells and histograms depicting CD38 expression between the two treatment conditions clearly demonstrate a significant decrease in CD38 expression in the residual tumor cells exposed to αCD38 antibody, whereas those in the IgG isotype condition retained their robust CD38 expression (Fig. 2C). Quantitative analysis of the percentage of CD45+ tumor cells expressing CD38 in each condition revealed a highly significant decrease in the cells treated with αCD38 antibody (*p* < 0.0001 by an unpaired *t* test) (Fig. 2D). To see if this decrease was transcriptionally regulated, we looked at *CD38* gene expression between these two sets of samples. We found that despite showing decreased surface protein CD38 expression, the CTCL cells exposed to αCD38 antibody had increased *CD38* gene expression (*p* < 0.0001) (Fig. 2E), while no increase in internalized CD38 expression was observed in permeabilized cells following in vitro incubation with daratumumab (Supplementary Fig. 2). In addition to increased *CD38* gene expression, the CTCL cells treated with αCD38 antibody also had significantly increased expression of known drivers of T-cell proliferation *B-catenin* (*CTNNB1)*, *TCF-7*, and *BCL6* (all *p* < 0.0001) [16] (Fig. 2E).

### Growth and migration of CD38-deficient CTCL cells

**Fig. 3:**
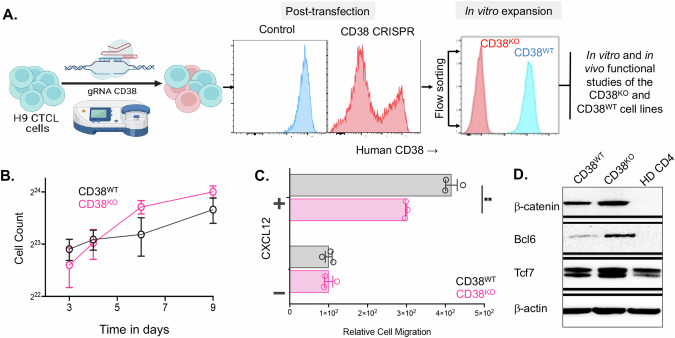
Comparison of growth, migration, and stemness protein expression in H9 CD38^WT^ and CD38^KO^ CTCL cell lines. **A** Schematic demonstrating development process of CD38^KO^ H9 CTCL cell line using CRISPR-Cas9, including flow cytometry showing post-sorting purity and CD38- status of CD38^KO^ line (figure made with Biorender). **B** CD38^KO^ CTCL cells showed no growth differences compared to CD38^WT^ (*p* = 0.22 by linear regression). **C** Migration assay comparing CD38^WT^ and CD38^KO^ CTCL cells with and without CXCL12 chemoattractant (*p* < 0.01 by unpaired *t* test). **D** Western blot of β-catenin, Bcl6, and Tcf7 protein levels with β-actin loading control comparing between H9 CD38^WT^ and H9 CD38^KO^ luciferase CTCL cell lines and healthy donor CD4 + T-cells (HD CD4) imaged via chemiluminescence with a FluorChem Imager and images processed with AlphaView software.

Having observed the loss of CD38 expression in CTCL cells following αCD38 antibody treatment, we aimed to delve deeper into the role of CD38 in the biology of CTCL cells. To achieve this, we employed CRISPR Cas-9 gene editing to create a CD38 knockout (CD38^KO^) CTCL cell line (H9). These knockout cells were then subjected to CD38 staining and sorted for purity (>99%). These purified cells were subsequently expanded and utilized for further analysis. Prior to each experiment, CD38 expression was confirmed via flow cytometry. The expanded cell line was employed for both in vitro and in vivo investigations, with mock transfected cell line serving as an experimental control, hereafter referred as CD38^WT^ (Fig. 3A). We monitored comparative cell growth over a span of nine days in culture and found no discernible differences in growth rates between the CD38^WT^ and CD38^KO^ lines (*p* = 0.22 by linear regression) (Fig. 3B). In order to compare differences in migration capacity between the CD38^WT^ and CD38^KO^ CTCL cells, we used a transwell migration assay with leukocyte chemoattractant CXCL12. We found that CD38^KO^ CTCL cells had significantly reduced migratory capacity compared to CD38^WT^ (*p* < 0.01) (Fig. 3C), despite the absence of detectable differences in CXCR4 expression between CD38^WT^ and CD38^KO^ CTCL cells (Supplementary Fig. 3). Additionally, given prior evidence suggesting that CD38 knockout in T-cells may enhance the expression of genes involved in oncogenic signaling [16], we compared β-catenin, Bcl6, and Tcf7 protein levels between CD38^WT^ and CD38^KO^ CTCL cells and healthy donor CD4 + T-cells. We observed significant increases in all three proteins in CD38^WT^ cells compared to healthy donor cells and further upregulation in CD38^KO^ cells compared to CD38^WT^ cells (Fig. 3D).

### Metabolic processes in CTCL cells lacking CD38

**Fig. 4:**
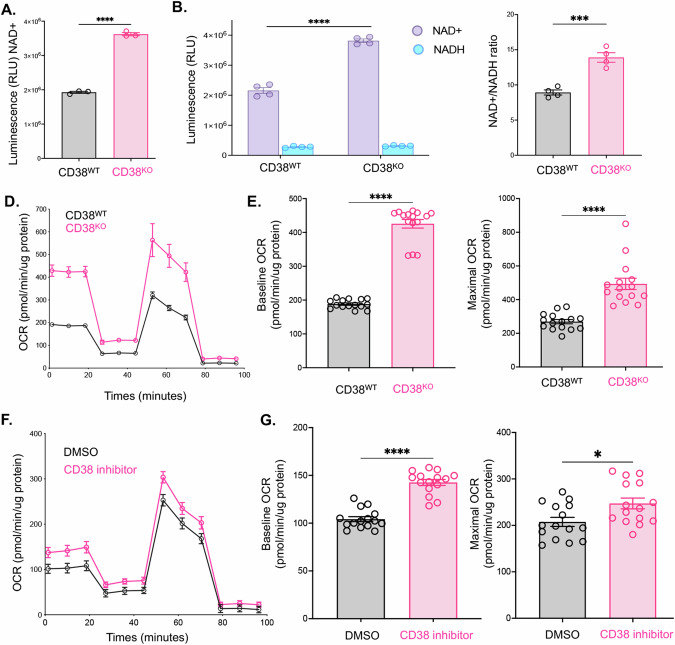
Enhanced metabolic activity in CD38^KO^ CTCL cell line and CD38^WT^ cells following CD38 inhibitor treatment. **A** Comparative assessment of total intracellular NAD+ and NADH levels between CD38^WT^ and CD38^KO^ CTCL cells using relative luminescence units (RLU) revealed significantly higher levels in CD38^KO^ cells (*p* < 0.0001 by unpaired *t* test). **B** Separate measurements of NAD+ vs NADH levels in CD38^WT^ and CD38^KO^ CTCL cells by RLU indicated significantly elevated NAD+ levels in CD38^KO^ cells (*p* < 0.0001 by unpaired *t* test). **C** Analysis of the NAD + /NADH ratio displayed a notable increase in CD38^KO^ cells (*p* = 0.0006 by unpaired *t* test). **D** Seahorse XF Cell Mito Stress Test assay compared oxygen consumption rate (OCR) between the CD38^WT^ and CD38^KO^ CTCL cell lines (normalized to pmol/min/µg protein). **E** Quantification of the average baseline normalized OCR as well as maximal OCR from the seahorse assay in panel D indicated a significant increase in both baseline and maximal OCR in CD38^KO^ cells compared to CD38^WT^ cells (baseline OCR showed a 2.26-fold increase, *p* < 0.0001; maximal OCR exhibited a 1.83-fold increase, *p* < 0.0001 as determined by the Mann–Whitney test). **F** Seahorse XF Cell Mito Stress Test assay compared OCR of CD38^WT^ cells treated with CD38 inhibitor compound 78c (0.5 µM overnight) or a DMSO control. **G** Quantification of baseline and maximal normalized OCR between CD38 inhibitor-treated cells and DMSO control cells demonstrated a significant increase in both baseline and maximal OCR in the CD38 inhibitor-treated cells (baseline OCR 1.38-fold increase, *p* < 0.0001; maximal OCR exhibited a 1.19 fold increase, *p* = 0.021 as determined by Mann–Whitney test).

Given CD38’s established role as an enzymatic metabolizer of NAD + , which is known to influence cellular respiration and metabolism [16], we conducted an investigation into the levels of NAD+ and NADH in CD38^KO^ CTCL cells using the NAD + /NADH-Glo assay (Promega). This assay employs luminescence as a measure of NAD/NADH substrate levels. The quantification of the combined intracellular NAD and NADH levels revealed significantly elevated amounts of these substrates in CD38^KO^ cells compared to CD38^WT^ cells (*p* < 0.0001 by unpaired *t* test) (Fig. 4A). To delve deeper into the metabolic differences, we employed a separate acid/base treatment and heating method to measure NAD+ and NADH levels individually. Our findings indicated notably increased levels of NAD+ in CD38^KO^ cells, with no significant difference in NADH between the two cell lines (*p* < 0.0001 by unpaired *t* test) (Fig. 4B). This observation translated to a significantly increased NAD+ to NADH ratio in CD38^KO^ cells (*p* < 0.0001 by unpaired t test) (Fig. 4C). To further assess metabolic distinctions between the CD38^WT^ and CD38^KO^ lines, we conducted an XFe24 Seahorse Mito Stress Test assay. Our results revealed increased cellular respiration, quantified by oxygen consumption rate (OCR; pmol/min/µg protein) in CD38^KO^ cells compared to CD38^WT^ cells (Fig. 4D). CD38^KO^ cells exhibited a significantly higher 2.26-fold increase in baseline OCR versus the CD38^WT^ (*p* < 0.0001) and maximal OCR was 1.83-fold higher in the CD38^KO^ cells compared to CD38^WT^ cells (*p* < 0.0001) (Fig. 4E). In addition, we treated the CD38^WT^ cells with a CD38 inhibitor (78c compound, Selleckchem) at 0.5uM overnight to observe its impact on cellular respiration. The CD38^WT^ cells treated with CD38 inhibitor exhibited increased OCR compared to those treated with a DMSO control (Fig. 4F). These results demonstrated a significant increase in both baseline and maximal OCR (baseline OCR 1.38-fold increase, p < 0.0001; maximal OCR 1.19 fold increase, *p* = 0.021 by Mann–Whitney test) (Fig. 4G).

### Aggressive in vivo proliferation of CD38^KO^ CTCL cells in contrast to CD38^WT^ cells

**Fig. 5:**
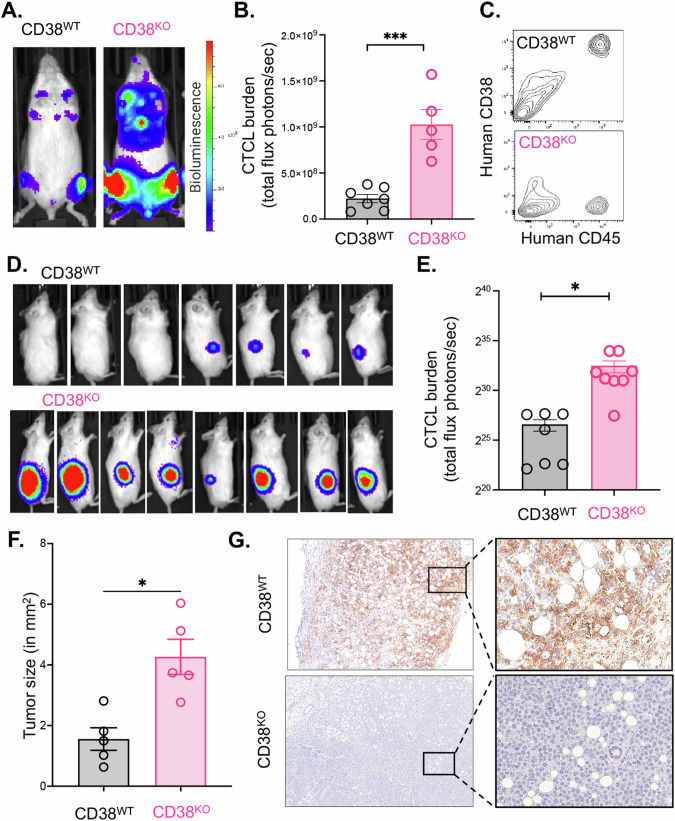
Enhanced in vivo growth of CD38^KO^ CTCL cells compared to CD38^WT^ cells. **A** Intravenously engrafted H9 CD38^WT^ and H9 CD38^KO^ CTCL cell lines in immunodeficient NOD Rag^−/−^γc^−/−^ (NRG) mice were monitored over time using an In Vivo Imaging System (IVIS; Perkins-Elmer) and representative images from day 18 post-engraftment are shown. **B** Quantification of the tumor cell luminescent intensity signal as measured by total flux (photons/second) in the CD38^WT^ and CD38^KO^ intravenous cohorts (CD38^WT^ = 2.2e8 photons/sec average total flux, *N* = 7; CD38^KO^ = 1e9 photons/sec, *N* = 5; *p* = 0.003 by Mann–Whitney test). **C** Flow cytometry analysis of human CD45+ tumor cells harvested the bone marrow of NRG mice post IV-engraftment and expansion of H9 CD38^WT^ and H9 CD38^KO^ luciferase CTCL cell lines. **D** IVIS images of tumor signal at 19 days post-subcutaneous flank engraftment of H9 CD38^WT^ and H9 CD38^KO^ luciferase CTCL cell lines in NRG mice. **E** Tumor cell burden as measured by total flux of subcutaneous CD38^WT^ and CD38^KO^ cells (CD38^WT^ = 1.01e8 photons/sec average total flux, *N* = 7; CD38^KO^ = 6.01e9 photons/sec, *N* = 8; *p* < 0.0001 by unpaired *t* test). **F** Flank tumors were dissected and photographed on a grossing board. Image J was used to measure tumor size in mm^2^ (*p* = 0.01 by Mann–Whitney test). **G** Dissected tumors were formalin fixed and paraffin embedded before being stained for human CD38 and imaged at original magnification x4 and x20 on a Cytation5 imager with Gen5 software.

Despite observing no disparities in in vitro growth between the CD38^WT^ and CD38^KO^ lines, but knowing there were metabolic differences between the lines, we were keen to investigate their behavior in an in vivo setting using a xenograft mouse model of CTCL. To enable monitoring of their progression over time, we introduced the firefly luciferase gene into both the CD38^WT^ and CD38^KO^ CTCL cell lines through lentivirus transduction. Subsequently, we intravenously engrafted CD38^WT^ and CD38^KO^ luciferase CTCL cells into immunodeficient NOD Rag^−/−^γc^−/−^ (NRG) mice and monitored their development over time using a luminescence in vivo imaging system (IVIS; Perkins-Elmer). Notably, the CD38^KO^ cells exhibited a significantly more aggressive engraftment, evident from the high-intensity (red) signal detected in the IVIS images (Fig. 5A). The intensity of bioluminescent signal can be quantified as total flux (photons/second). We observed that the average total flux of the CTCL cell burden was significantly higher in the CD38^KO^ mice (*N* = 5) compared to their CD38^WT^ counterparts (*N* = 7) (*p* = 0.003 by Mann–Whitney test) (Fig. 5B). Importantly, when we collected bone marrow from these mice at the end of the study and stained the cells for human CD45+ and human CD38+ for flow cytometry analysis, the CTCL tumor cells had maintained their respective CD38+ and CD38- expression statuses (Fig. 5C).

In addition to evaluating these cells in the context of intravenous engraftment, we sought to compare the behavior of CD38^WT^ and CD38^KO^ cells in a subcutaneous setting. Given that CTCL primarily affects the skin, we were interested in understanding whether engrafting these cells into a subcutaneous environment would influence our observations. To investigate this, we subcutaneously transplanted CD38^WT^ and CD38^KO^ luciferase expressing CTCL cells into the flanks of NRG mice and closely monitored the progression of tumors over time, both through visual examination and IVIS imaging. Remarkably, CD38^KO^ tumors exhibited significantly more aggressive growth compared to CD38^WT^ tumors. The CD38^KO^ tumors not only emitted a stronger luminescent signal but were also palpably larger (Fig. 5D). Quantitative analysis of luminescent intensity revealed a substantial increase in the CD38^KO^ tumors compared to CD38^WT^ tumors (p < 0.0001 by unpaired *t* test) (Fig. 5E). Furthermore, upon dissection, we captured images of the tumors and employed Image J to calculate their sizes, confirming that the CD38^KO^ tumors were significantly larger than the CD38^WT^ counterparts (Fig. 5F). To further validate the CD38 phenotypes, we fixed these tumors in formalin and paraffin embedded them for immunohistochemistry. Staining for human CD38 demonstrated robust expression in the CD38^WT^ tumors, whereas it was entirely absent in the CD38^KO^ tumors (Fig. 5G).

### Elevating CD38 expression on CTCL cells in vitro using panobinostat results in a survival advantage when combined with αCD38 antibody in vivo

**Fig. 6:**
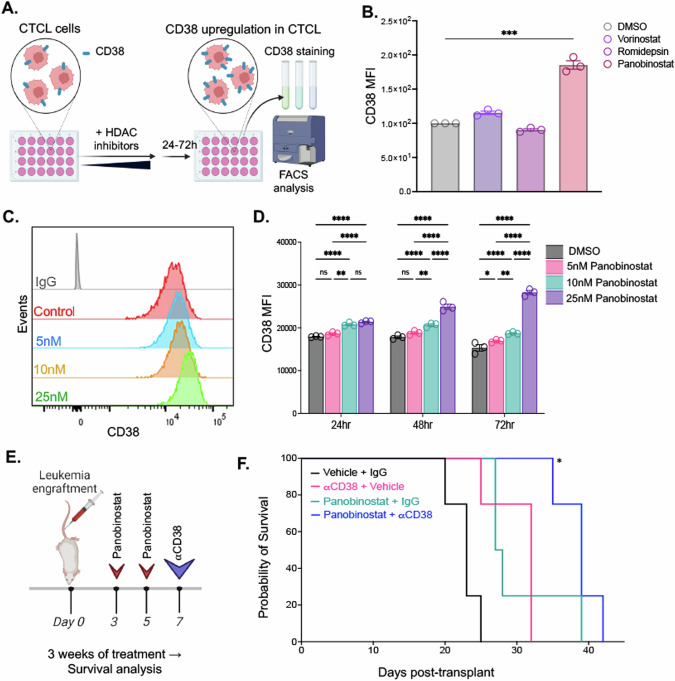
Panobinostat upregulates CD38 expression on H9 CTCL cells and enhances survival in a mouse model of CTCL in combination with αCD38 antibody immunotherapy. **A** CTCL cells were treated with one of several histone deacetylase inhibitor agents followed by staining for CD38 and subsequent analysis via flow cytometry using a BD LSRFortessa (figure made with Biorender). **B** The mean fluorescent intensity of CD38 expression on CTCL cells treated for 72 h with either 1 μM vorinostat, 1 nM romidepsin, 25 nM of panobinostat, or DMSO control (panobinostat vs DMSO *p* < 0.001 by unpaired *t* test). **C** Representative histograms displaying CD38 expression at 72 h for increasing doses of panobinostat (5 nM, 10 nM, and 25 nM) compared to an isotype control. **D** Median fluorescence intensity (MFI) of CD38 expression on H9 cells treated with increasing doses of panobinostat for across multiple time points (24 h, 48 h, and 72 h) (max 85% increase in CD38 expression with 25 nM panobinostat vs. DMSO at 72 h; *p* < 0.0001 by 2way ANOVA). **E** Experimental design and treatment regimen for the in vivo combination αCD38 antibody and panobinostat in mice engrafted H9 luciferase CTCL cells. The animals were divided into four age and sex-matched experimental groups (*N* = 4 for all groups): Vehicle and IgG; Vehicle and αCD38 antibody; Panobinostat and IgG; and Panobinostat and αCD38 antibody. Panobinostat (20 mg/kg) and vehicle (2% DMSO, 48% PEG300, 2% Tween80, and 48% ddH2O) were administered via IP injection twice a week, while αCD38 antibody (daratumumab 100 mg/kg) and IgG isotype control (0.8 mg/kg) were administered subcutaneously once a week for three weeks. Mice were monitored and survival was tracked (figure made with Biorender). **F** Survival curve depicting the outcomes of the four experimental groups in the αCD38 antibody and panobinostat combination study (vehicle/IgG median survival 23 days; vehicle/ αCD38 antibody median survival 32 days; Panobinostat/IgG median survival 27.5 days; combination median survival 39 days; *p* = 0.01 by log-rank test).

As has been previously demonstrated in the context of MM, it has been shown that various pharmacologic agents can be combined with αCD38 antibody to augment its efficacy by increasing CD38 expression on target tumor cells [6, 8, 9]. To explore the potential applicability of this approach in the context of CTCL, we conducted in vitro experiments with CD38^WT^ CTCL cells (Fig. 6A). Cells were treated with one of three agents (1uM Vorinostat, 1 nM Romidepsin, or 25 nM panobinostat) for 72 h before evaluating the intensity of CD38 expression on the cells’ surface. We found the greatest increase in CD38 surface expression with panobinostat when compared to DMSO control (*p* < 0.001) (Fig. 6B). CD38^WT^ CTCL cells were then exposed to escalating doses of panobinostat (5 nM, 10 nM, and 25 nM) over several time intervals, after which we measured their CD38 expression levels using flow cytometry. We found a reliable dose-dependent increase in CD38 expression at each of the doses tested (Fig. 6C). Our findings indicated a consistent and dose-dependent increase in CD38 expression at each of the tested time points (Fig. 6D). Furthermore, this dose-dependent increase in CD38 was statistically significant across all three time points examined, with the highest increase occurring at 72 h with 25 nM panobinostat compared to the DMSO control (*p* < 0.0001 as determined by 2-way ANOVA) (Fig. 6D). These results underscore the reliable ability of panobinostat to induce CD38 expression in CTCL cells in vitro.

To assess whether this elevated CD38 expression in tumor cells conferred any therapeutic benefits, we conducted a combination therapy study involving αCD38 antibody and panobinostat using our mouse model of CTCL. We engrafted NRG mice with CD38^WT^ CTCL luciferase cells intravenously, categorizing them into one of four experimental groups: Vehicle and IgG; Vehicle and αCD38 antibody; panobinostat and IgG; and panobinostat and αCD38 antibody (*N* = 4 in all groups). Panobinostat and vehicle were administered twice a week, while αCD38 antibody and IgG isotype control were given once a week for three weeks, during which the mice were monitored and tracked for their survival (Fig. 6E). Tumor burden, assessed using IVIS imaging, revealed changes during treatment, with quantification provided in Supplementary Fig. 4A, B, and a statistically significant survival advantage was observed in mice receiving combination therapy with αCD38 and panobinostat compared to those that received αCD38 antibody alone (*p* = 0.01 by log-rank test) (Fig. 6F). Additional qPCR analysis of tumor cells from the mouse bone marrow revealed a significant increase in relative *CD38* expression in the panobinostat treatment groups compared to αCD38 antibody alone, highlighting the impact of panobinostat on enhancing CD38 gene expression in vivo (Supplementary Fig. 4C).

## Discussion

Treating CTCL poses challenges due to relapse and resistance. Identifying targeted therapy is crucial. Building on the success of CD38 targeting in mature T-cell neoplasms, we sought to apply this strategy to CTCL [3]. Notably, daratumumab, an αCD38 antibody, significantly reduced CTCL tumor burden by 84%, but residual cells persisted, impacting post-treatment tumor growth. This study reveals characteristics of CD38 antibody resistant CTCL cells in vivo, emphasizing the therapy’s promise in conditions with elevated CD38 expression, such as hematological malignancies [17]. Crucially, for CTCL targeted therapies, we employed CRISPR-Cas9 to create CD38 knockout cell lines, studying its role in resistant cells in vitro and in vivo. In a xenograft model, we innovatively combined a CD38-inducing agent with CD38-targeting antibodies, revealing insights into CD38’s significance in CTCL pathology and suggesting novel treatment strategies.

These findings extend our prior research on elevated CD38 levels in mature T-cell neoplasms and align with recent studies demonstrating CD38 expression in tumor cells of aggressive, treatment-resistant CTCL patients [3, 15]. Previous investigations on CD38 expression in CTCL have been limited and somewhat inconsistent, particularly in Sézary Syndrome (SS) cells, which exhibited intermediate CD38 staining in treatment-resistant, aggressive CTCL subtypes [15, 18]. Some studies suggested a predominantly CD38-negative phenotype in SS patients, although a small cohort of CD38 + SS patients with strong CD38 expression was also noted [19–21]. Nevertheless, the diverse levels of CD38 expression observed among different CTCL subtypes and individuals highlight the necessity for further research to elucidate the connection between CTCL subtypes and the prognostic significance of CD38 expression.

In vitro, the αCD38 antibody has been shown to engage antibody-dependent cellular phagocytosis and antibody-dependent cellular cytotoxicity mechanisms, effectively eliminating primary patient CTCL cells [15]. This translational potential was exemplified in a case study involving a patient with concurrent multiple myeloma (MM) and SS who received daratumumab. This patient exhibited reduced CTCL tumor cell counts and improvements in skin erythroderma [15]. Nonetheless, while our findings indicated the effectiveness of CD38-directed antibody therapy in eliminating tumor cells in vivo, the residual cells that persisted and evaded treatment showed a decline in CD38 expression on their surfaces. This observation aligns with previous reports on the effects of αCD38 antibody treatment in MM, where it leads to CD38 internalization and a subsequent reduction in surface CD38 expression [7, 22]. Our data indicate that CD38^KO^ cells exhibit heightened metabolic activity and demonstrate more aggressive tumor growth in vivo. These results correspond with earlier research involving CD38^KO^ mice, which also displayed increased levels of NAD+ and higher oxygen consumption rates in CD4 + T-cells compared to CD38^WT^ mice [16]. The elevated expression of “stemness” genes and proteins such as Tcf7, Bcl6, and β-catenin in CD38^KO^ cells supports the concept of a CD38-NAD+ signaling pathway that promotes cellular persistence and pluripotency, which could be problematic in malignant CTCL cells [16, 23]. Molecularly, the overexpression of Tcf7, Bcl6, β-catenin, and possibly other genes can contribute to cancer progression and the risk of relapse. However, it’s crucial to acknowledge the influence of factors like the tumor microenvironment on long-term therapeutic responses. These findings strongly support the need for additional investigations and potential clinical trials to explore the therapeutic benefits of targeting CD38 in patients with CD38 + CTCL.

Our in vivo results strongly support the use of daratumumab, a CD38-targeting antibody, in combination with agents that stimulate CD38 expression. This combination approach has demonstrated its efficacy in other hematological malignancies. For example, in multiple myeloma (MM), combining daratumumab with other agents like proteasome inhibitors (e.g., bortezomib), immunomodulatory drugs (e.g., lenalidomide), or corticosteroids has shown significant clinical benefits [24, 25]. These combinations have led to increased response rates, and improved outcome in MM patients. Similarly, in Acute Lymphoblastic Leukemia (ALL), combining CD38 antibodies like daratumumab with chemotherapy regimens has yielded promising outcomes, particularly for patients with high CD38 expression [26]. This approach shows better patient outcomes. Immune cells likely enhance conventional chemotherapy’s cytotoxic effects, but targeted agents may offer a higher therapeutic index if better tolerated. Evaluating targeted agents solely as monotherapies may underestimate their benefits, especially in heterogeneous diseases like CTCL. Our research emphasizes the importance of empirically testing drug combinations in rigorous in vivo models. Panobinostat effectively increases CD38 expression on CTCL cells and combining it with αCD38 antibody significantly improves survival outcomes compared to daratumumab or panobinostat alone. In summary, our results highlight the complex role of CD38 in CTCL biology and stress the importance of identifying optimal drug combinations when used alongside CD38 antibodies for superior outcomes in CTCL patients.

## Supplementary information

Supplementary Clean

## Data Availability

RNA sequencing datasets used in this study have previously been published as cited above and are publicly available. Data that were generated in the experiments in support of the conclusions of this study are available from the corresponding author upon reasonable request.
