# Supplementary material for: CD38 in the pathobiology of cutaneous T-cell lymphoma and the potential for combination therapeutic intervention
Source: Leukemia. 2025 Mar 8;39(5):1146–56. doi: 10.1038/s41375-025-02551-4 (PMC12055602; doi:10.1038/s41375-025-02551-4)
Supplement: Supplementary file 1 — Supplementary Clean [file 41375_2025_2551_MOESM1_ESM.docx]

**Supplementary Materials and Methods:**

***Cell Culture and Flow Cytometry:*** Cells were stained in 1% FBS in PBS FACs buffer and analyzed on BD LSRFortessa or BDFACSAriaII flow cytometers using BD FACSDiva or FlowJo software (BD Biosciences). For CD38 enhancing drug study, H9 CTCL cells were treated for 72 hours with 1uM vorinostat (MCE Cat# HY-10221); 1nM romidepsin (MCE Cat# HY-15149); or 25nM panobinostat (MCE Cat# HY-10224) before staining for flow cytometry. In panobinostat dosing experiments, cells were exposed to three doses (5nM, 10nM, and 25nM) for 24, 48, and 72 hours before flow cytometry staining. These experiments were replicated twice with at least three technical replicates per condition.

***Gene Expression Analysis:*** For single cell RNA-seq gene expression the Seurat R package (v4.3.0) was employed to filter the data, selecting cells with unique gene counts between 200 and 6000, and mitochondrial DNA content ranging from 5% to 15%. After filtering, a total of 46,785 cells were included in the analysis. Log-normalization was applied for scaling and normalization of the data. The 2000 most variable genes across the dataset were identified and utilized for principal component analysis (PCA). Cluster-specific genes were determined using the Wilcoxon Rank Sum test, focusing on genes with expression levels above 25% and a log fold-change exceeding 0.25. qPCR analysis was performed on a Quant Studio 3 (Applied Biosystems) to compare the relative expression of genes in CTCL tumor cells isolated from the bone marrow of mice treated with either αCD38 antibody (n=4), panobinostat (n=4), or both (n=3). RNA was isolated from tumor cells in the bone marrow, which were fresh frozen at -80degC and processed via Total RNA Purification Plus (Norgen Biotek, ON, Canada, 48300) following kit directions without modifications. Nucleic acid quantification and contamination was assessed via NanoDrop (Thermo Scientific, 840274100). cDNA was made with the ProFlex PCR System (Applied Biosystems, 4484073) using 1 ug of RNA in a 20 ul reaction volume with SuperScript IV VILO Master Mix (ThermoFisher Scientific, CA, USA, 11756050) and temp and time reaction conditions as recommended. cDNA was analyzed on system using Taqman Fast Advanced Master Mix (ThermoFisher, 4444964) and assays for *CD38* (Hs01120071_m1) and *18S* (Hs99999901_s1) as reference gene. Ct values were generated using the default system settings and analyzed using 2^-ΔCt method. All qPCR analyses had at least three technical replicates per sample per experiment.

***CTCL Xenograft Mouse Models:*** All experiments were conducted in a randomized manner to ensure unbiased results. This approach eliminated prior bias and contributed to the reliability and validity of the findings. The experiments were not blinded, but outcome measurements and analyses were conducted using predefined criteria to minimize bias^3^. Mice were only excluded from analysis in the case of failure of engraftment as determined by a persistent lack of IVIS signal over time that was inconsistent with their cohort. Subcutaneous tumors were dissected, photographed, and measured using ImageJ. Bone marrow was harvested from femur and tibia and processed for flow cytometry analysis.

***Immunoblotting:*** CD38^WT^ and CD38^KO^ cells were lysed for protein quantification and normalized using Bio-Rad Protein Assay Dye Reagent Concentrate (Biorad, #5000006). Samples were denatured and prepared with 4x Laemmli Sample Buffer (BioRad, #1610747) for gel electrophoresis (BioRad). Gels were transferred to PVDF membranes with the iBlot2 (Invitrogen) system. Primary antibodies included Bcl6, Tcf-1/Tcf7, β-catenin, and β-actin (all from Cell Signaling Technology), along with an anti-rabbit secondary antibody (CST, #7074S). Blots were imaged using a FluorChem Imager and processed with AlphaView software (Biotechne, Minneapolis, MN).

***Histology:*** Human skin FFPE biopsies collections were approved under an Institutional Review Board approved protocol in accordance with the Declaration of Helsinki with informed consent obtained from all subjects. The samples were sectioned and stained with an anti-human CD38 antibody (Cell Signaling #51000). H9 CD38^WT^ and CD38^KO^ CTCL cell line tumors were grown in NRG mice via subcutaneous injection of 2 million cells. These tumor samples were formalin-fixed and paraffin-embedded, then stained with an anti-human CD38 antibody. Imaging was conducted at 4x and 20x magnification using a Cytation5 imager and Gen5 3.05 software (Biotek, Winooski, VT).

***Sample Size and Statistical Methods***: Each experiment was performed at least three times with at least three technical replicates. *In vivo* experiments were performed at least three times with cohort size of at least three animals per cohort. Effect size estimates were based on previous studies (PMID: 36989058), and variability was derived from pilot data, assuming a normal distribution. We have represented the variation within each group in the figures and provided detailed explanations in the figure legends. The data are displayed as mean ± SEM to show the variability across biological or technical replicates. Sample sizes (n) are also included in the legends for each data. For animal experiments, the sample size was determined based on prior studies and experience to detect differences, considering variability and ethical guidelines to minimize animal use, though no formal statistical calculations were used. For statistical analysis of data, student t-test and Mann-Whitney tests were performed for two-group comparisons. Variance was similar between groups. ANOVA was used when comparing three or more groups. Kaplan-Meier survival curves were used to analyze survival probability, while log-rank test was used to compare survival curves.

A.

B.

**Supplementary Figure 1: Dosage titration of hIgG1k isotype control shows no difference in tumor burden or survival over time between 100mg/kg or 0.8mg/kg.** (A) hIgG1k isotype control antibody was dose-titrated for experimental practicality using immunodeficient NOD Rag^-/-^gc^-/-^ (NRG) mice engrafted with H9 CD38^WT^ luciferase CTCL cells and randomly assigned to age and sex-matched treatment groups (n=3 mice per group). CTCL luciferase tumor model mice were treated with either 100mg/kg or 0.8mg/kg isotype control and tumor burden was monitored over time via an in vivo imaging system (IVIS). CTCL tumor cell burden was quantified as total flux photons/sec (p>0.05, 2-way ANOVA). (B) Survival probability of subjects was tracked over time in both the 100mg/kg and 0.8mg/kg hIgGk1 isotype conditions (Kaplan-Meier survival curve).

**Supplementary Figure 2: No increase in CD38 expression observed in permeabilized CTCL tumor cells post-daratumumab treatment.** H9 CD38^WT^ luciferase CTCL cells were treated with daratumumab αCD38 antibody (100ug/mL) or IgG isotype control (100ug/mL) for 1 hour. Tumor cells were permeabilized and stained for CD38 according to previously published protocol^7^. Cells were analyzed for CD38 expression via flow cytometry using a BDFACS Aria and FlowJo analysis software. ​


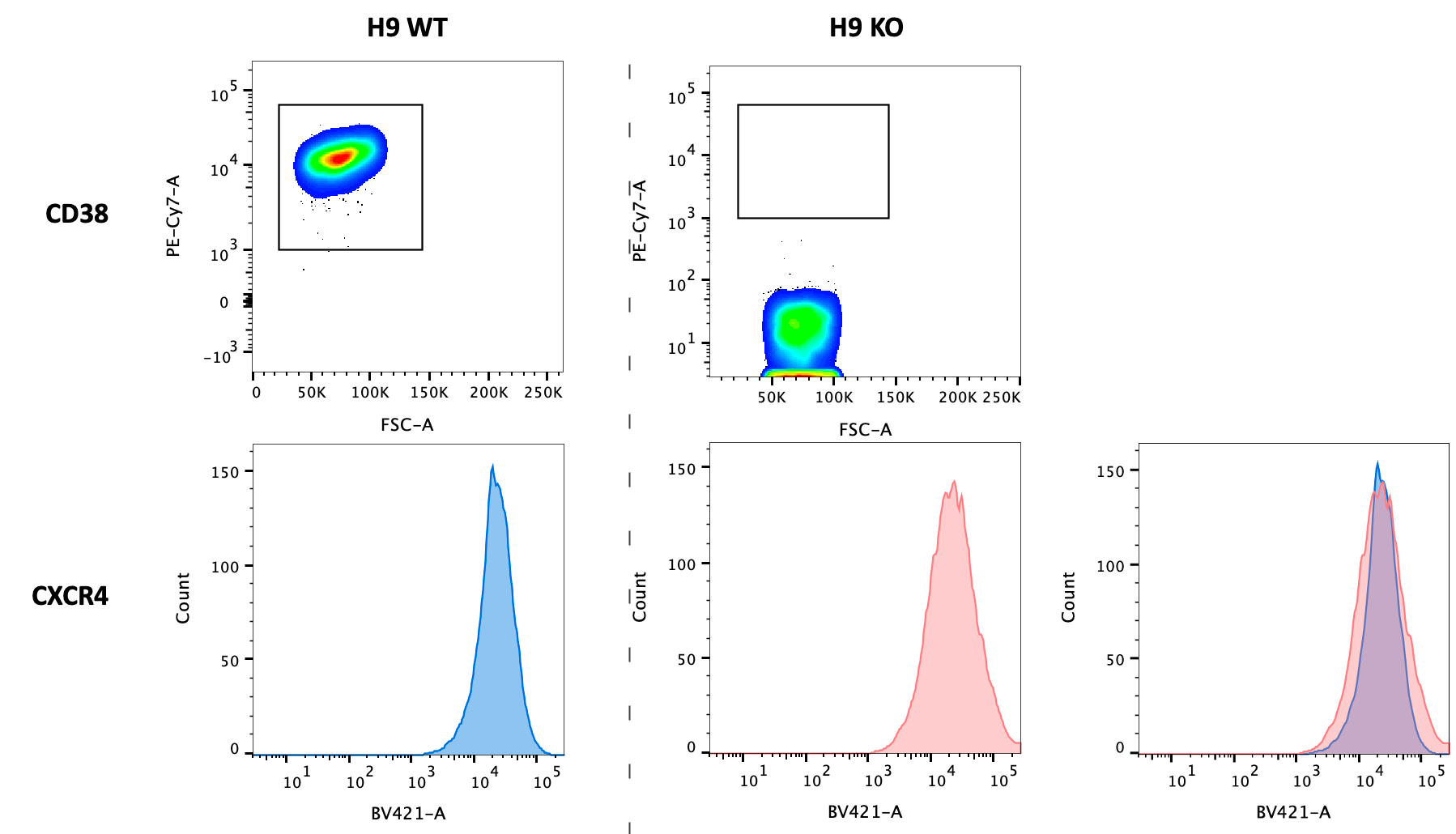


CD38^WT^

CD38^KO^

CD38

CD38

Count

CXCR4

CXCR4

CD38^WT^

CD38^KO^

CD38^WT^

CD38^KO^

CXCR4

**Supplementary Figure 3: No differences observed in CXCR4 expression between CD38^WT^ and CD38^KO^ H9 CTCL cells.** ​CTCL H9 CD38^WT^ and CD38^KO^ tumor cell lines were stained for CD38 and CXCR4. They were analyzed for CD38 expression (upper panels) as well as for CXCR4 expression (lower panels) via flow cytometry.

**Supplementary Figure 4: CD38 expression increases in CTCL cells exposed to daratumumab, while tumor burden decreases in mice treated with combination daratumumab and panobinostat therapy.** (A) Immunodeficient NOD Rag^-/-^gc^-/-^ (NRG) mice were intravenously engrafted with H9 CD38^WT^ luciferase CTCL cells and randomly assigned to one of four age and sex-matched treatment groups (N=4 for all groups): Vehicle (2% DMSO, 48% PEG300, 2% Tween80, and 48% ddH2O) and IgG (0.8mg/kg); Vehicle and αCD38 (daratumumab 100mg/kg) antibody; Panobinostat (20mg/kg) and IgG; and Panobinostat and αCD38 antibody. Mice were monitored via serial *in vivo* imaging (IVIS; Perkins-Elmer) and survival tracking. Representative IVIS images of tumor signal on day 24 post-engraftment in each of the four treatment conditions are shown. (B) Tumor burden quantified from total flux signal (photons/second) in each of the four treatment conditions (**p=0.0086, *p=0.0314, **p=0.0046 by one-way ANOVA test). (C) CTCL tumor cells were harvested from the bone marrow of the above mice treated with daratumumab alone, panobinostat alone, or the combination of panobinostat/daratumumab. Relative expression of *CD38* was analyzed by qPCR (*p=0.047 by paired t-test; replicated once with four biological replicates).
